# A study on improving the prediction accuracy of cold forging die life based on quantitative evaluation of phosphate film damage

**DOI:** 10.1038/s41598-023-43400-7

**Published:** 2023-09-30

**Authors:** Young Ho Seo

**Affiliations:** Automotive Materials & Components R&D Group, KITECH, Cheomdan-Venturero 108, Gwangju, 61007 Korea

**Keywords:** Engineering, Materials science

## Abstract

In designing the multi-stage cold forging process, an important factor along with formability is the die life cycle. As the die life cycle is closely related to the integrity of the phosphate film coating on the surface of the material, this study presents a method for predicting the die life cycle considering damage to the phosphate film coating. First, the correlation between the phosphate treatment conditions (phosphate treatment solution concentration and temperature) and the film layer weight was discussed. Afterwards, the behavior of the friction coefficient according to the damage of the phosphate film was predicted through repeated frictional tests. The behavior of the coefficient of friction was mainly divided into three areas and correlated with the film weight. By applying these results to the automobile engine bolt forging process, a method for performing precise simulation considering the damage to the phosphate film was presented. In addition, a method for predicting the quantitative limit life of the die based on precise simulation results with high accuracy was presented.

## Introduction

**Figure 1:**
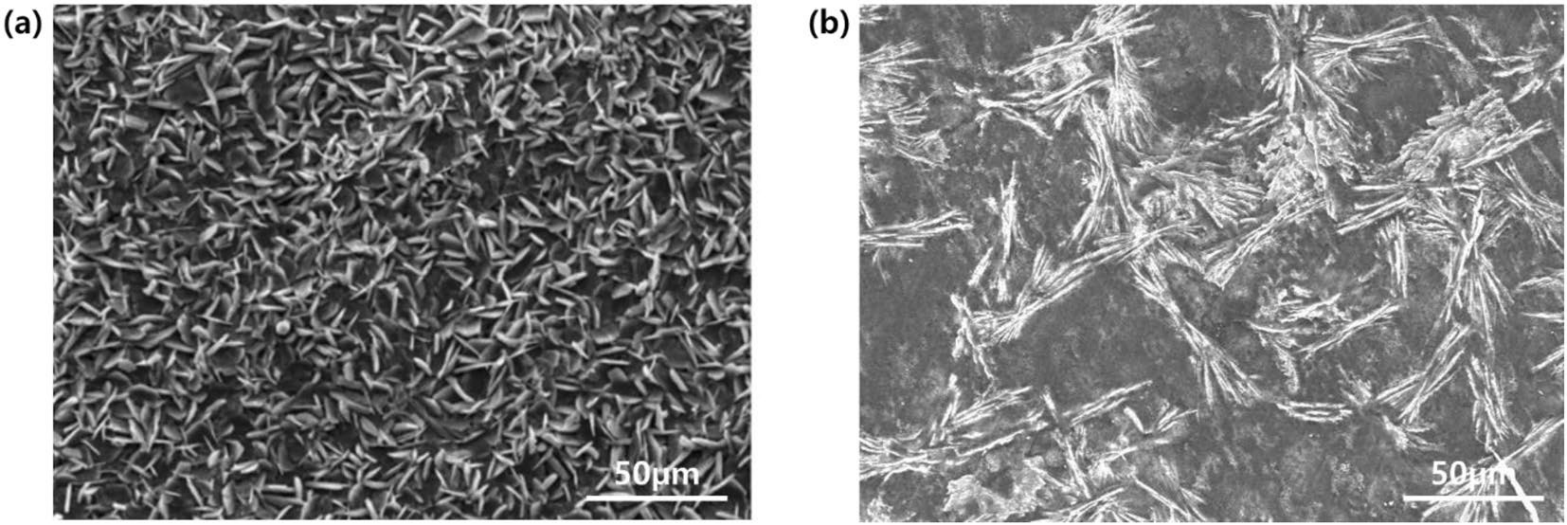
Phosphate crystallite size for (**a**) corrosion resistance and (**b**) lubrication purposes.

Wire rods are mainly used in the cold forging process, and are shipped from steel makers in the form of semi-finished products. Afterwards, spheroidizing and low annealing heat treatment are performed to soften the material. In addition, to acquire the required lubrication performance of the material’s surface, it is treated with a phosphate film coating, and finally, the appropriate diameter of the material is secured through the wire drawing process. Phosphate coating treatment creates a compound film with high adhesion and stability on the metal surface^1^. It is a surface treatment method to protect metals from corrosion^2^ or friction/abrasion^3^ by using the physical or chemical properties of the compound created on the metal surface. In general, phosphate treatment for painting, which is performed for the purpose of preventing corrosion of metal surfaces, improves corrosion resistance more as the grain size decreases^4,5^. On the other hand, phosphate treatment for lubrication has a relatively large grain size and a large surface area, enabling it to offset the frictional force between the material and the die surface^6^. As shown in Fig. 1, the grain sizes of phosphates are different depending on the purpose.

Phosphate coating methods applied to cold forming are classified into phosphate-ferrous, phosphate-manganese, phosphate-zinc, and phosphate-zinc calcium^7^. Of these, there are more concerns related to lowering of corrosion resistance with phosphate-ferrous based coating than with phosphate-manganese based film, but phosphate-ferrous based coating is widely used to this day because it can perform surface rust removal and film treatment at the same time. The general treatment process of phosphate film coating goes through processes of degreasing-pickling-chemicalization-neutralization-lubrication-drying^6,8,9^. The main components of the chemical conversion solution that creates the phosphate film are phosphoric acid, zinc, and nitric acid, and the resulting chemical conversion film is composed of zinc-phosphate (hopeite) and zinc-ferrous-phosphate (phosphophyllite). Ferrous corrosion by free phosphoric acid occurs in the initial stage of the chemical reaction. Through an increase in pH at the diffusion layer, a chemical conversion film is deposited on the metal surface by shifting the chemical equilibrium^10,11^. The components of the chemical conversion film thus formed are as follows.

$$ {\text{zinc-phosphate }}\left( {{\text{hopeite}}} \right) \, :{\text{Zn}}_{{3}} \left( {{\text{PO4}}} \right)_{{2}} \cdot {\text{4H}}_{{2}} {\text{O}} $$

$$ {\text{zinc-ferrous-phosphate }}\left( {{\text{phosphophyllite}}} \right):{\text{ Zn}}_{{2}} {\text{Fe}}\left( {{\text{PO}}_{{4}} } \right)_{{2}} \cdot {\text{4H}}_{{2}} {\text{O}} $$

**Figure 2:**
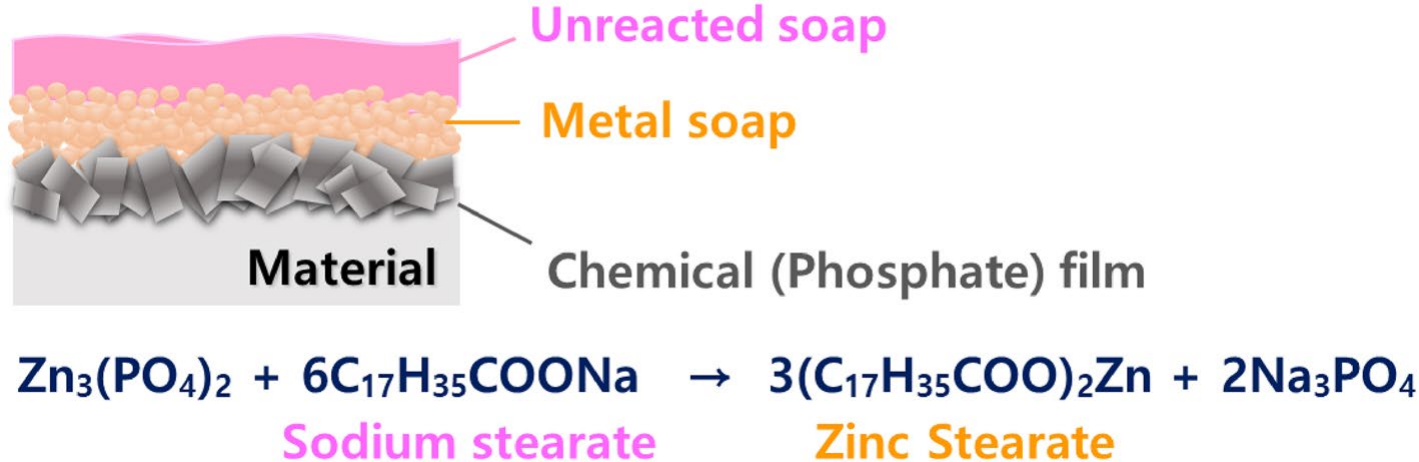
Structure of phosphate lubrication system.

As shown in Fig. 2, by dissolving the chemical conversion film in the lubricating solution, a metal soap layer (sodium stearate) is formed on the chemical film layer. Zinc stearate that has not reacted with the chemical conversion film remains as an unreactive soap layer. The chemical conversion film is highly involved in the film’s adhesion to the material’s surface, and the metal soap and unreacted soap layer, which are lubricating film layers, play a role in reducing friction between the material and the die surface.

The cold forging process is a method of securing a desired shape by compressing and deforming a material under a high load at room temperature. It is possible to obtain high shape accuracy, but due to the high forming load, seizure often occurs on the surface of the material and die^12,13^. This is a direct cause of loss, such as the occurrence of forming defects, increase in manufacturing cost, and decrease in productivity. Phosphate film coating should first be performed in order to inhibit seizure between the material and the die, but as the forging process progresses, the coating may become damaged or lost, so that the original performance is not achieved. As the multi-stage cold forging process progresses, quantitative evaluation of the performance of the phosphate film coating is required, as well as analysis of the effect of the film’s condition on the process and die conditions.

The repeated frictional test has been the one most widely used to define the mechanical properties of phosphate film coatings. In 1990, Bricout defined the correlation between the phosphate film and the coefficient of friction by rotating the test specimen while applying a constant vertical load^14^. In 2010, Bay proposed the ring test and double cup extrusion test as phosphate film evaluation methods^3^. In 2015, an ironing test method that substantially increases the surface area of the material was adopted to simulate the harsh environment in the multi-stage cold forging process^15^. However, no previous research has been conducted on the quantitative damage evaluation of phosphate film coating, the application of the friction coefficient for each forging step in connection with this, the process evaluation according to the deterioration of phosphate film performance, and the correlation with the cold forging die life cycle. In this study, the damage to the phosphate film was quantitatively analyzed. This was applied to the actual multi-stage cold forging process by deriving a correlation with the friction coefficient. In addition, by considering the damage to the phosphate film, the die limit life cycle can be predicted with higher accuracy.

## Phosphate treatment of specimens

In this study, the repeated frictional test was adopted as a method to simulate the gradual damage to the phosphate film on the material’s surface that occurs in the multi-stage cold forging process. The cold forging process of automotive steering parts is divided into an extrusion process that does not undergo much plastic deformation and a heading process that greatly occurs plastic deformation. Even in the stage dominated by extrusion deformation, the forming load reaches 5 to 20 tonf, and the forming load in the stage dominated by plastic deformation occurs more than 100 tonf. Backward extrusion can be considered as a friction test method for realizing high forming load conditions. However, the purpose of this study is to evaluate the gradual damage of the phosphate coating in a multi-stage process. Evaluation methods such as backward extrusion are not easy to cause gradual damage, and have the disadvantage of designing and manufacturing various molds. Therefore, a cyclic friction test was adopted, and a high forming load condition was induced with a large number of repetitions.

34CrMo4, which is most widely used in manufacturing automobile steering parts, was selected as the target material^16,17^. The diameter of wire rods manufactured as semi-finished products varies from 6 to 45 mm, and the phosphate film goes through the final drawing process to secure adhesion to the metal surface. In order to conduct a repeated frictional test of a 20.9 mm diameter 34CrMo4 material, a flat section of the surface treated with phosphate must be obtained. To this end, a load of a certain level or more is applied in the vertical direction of the cross-sectional area of the material. During this process, the surface of the material is subjected to tensile force, and the phosphate film is also deformed. Since the mechanical properties of the initial phosphate film change, a new phosphate film was created after removing the existing phosphate film after pressing with a vertical load.

**Figure 3:**
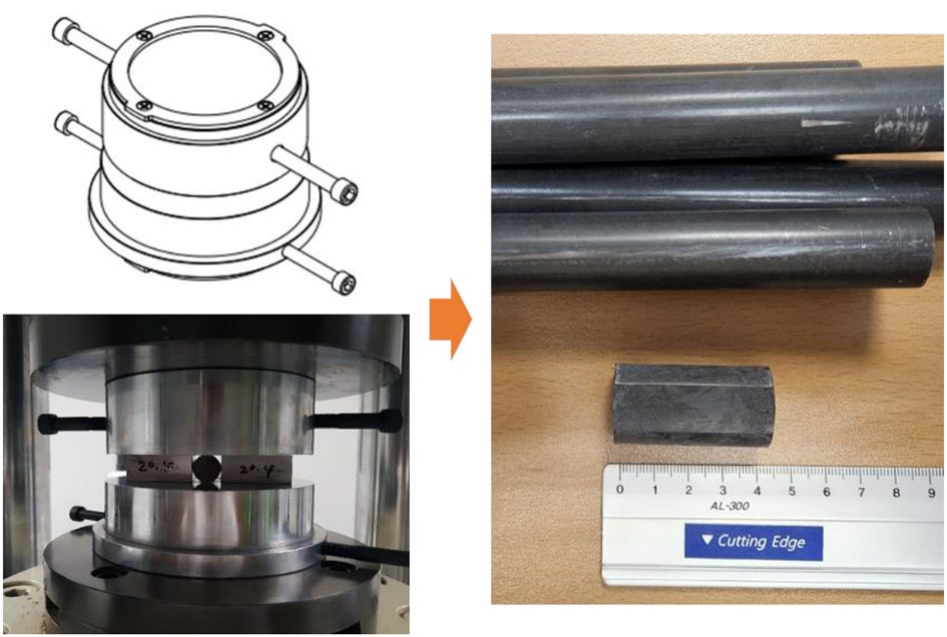
Flattening process of round bar material.

Vertical load was applied with the same stroke to all specimens using the compression jig manufactured as shown in Fig. 3. After cutting the 34CrMo4 material into 40 mm lengthwise, a test specimen having a flat section with a width of 10 mm was made using a 50-ton multipurpose forming machine. The existing phosphate film was removed through the washing-degreasing-pickling process. In the phosphate treatment step, the concentration and treatment temperature of the solution were set to three levels, respectively, and the treatment time was kept constant at 10 min. Phosphating process conditions in each step are described in detail in Table 1^18^. All phosphate treatment was based on Nippon Parkerizing Company's process standards, and descriptions of detailed chemical solutions are omitted due to security policy. The factors affecting the formation of the phosphate film are the concentration of the phosphate solution and the treatment temperature. The phosphate treatment standards are 131.6 g/L (50 pt) and 80 °C, and the upper and lower limits are shown in Table 1. In this study, the research scope was limited within the phosphate treatment standard. A total of 45 EA test specimens were prepared, 5 EA for each phosphate solution concentration and treatment temperature condition. A test specimen of 1 EA for each condition was separately classified and used for measuring the weight of the phosphate film coating.

**Table 1:** Phosphating process summary.

Process	Chemical liquid		Concentration [g/L]	Temp. [°C]	Time [min]
Degreasing	FC-4461		25	60	10
Pickling	HCL		–	–	1
Phosphating	DP-CH200M + AJ-4770	Lower	78.9 (30pt)	65	10
		Standard	131.6 (50pt)	80	
		Upper	157.8 (80pt)	90	
Neutralization	PL-27		1.9	60	3
Lubrication	DL-301		27	85	3

**Figure 4:**
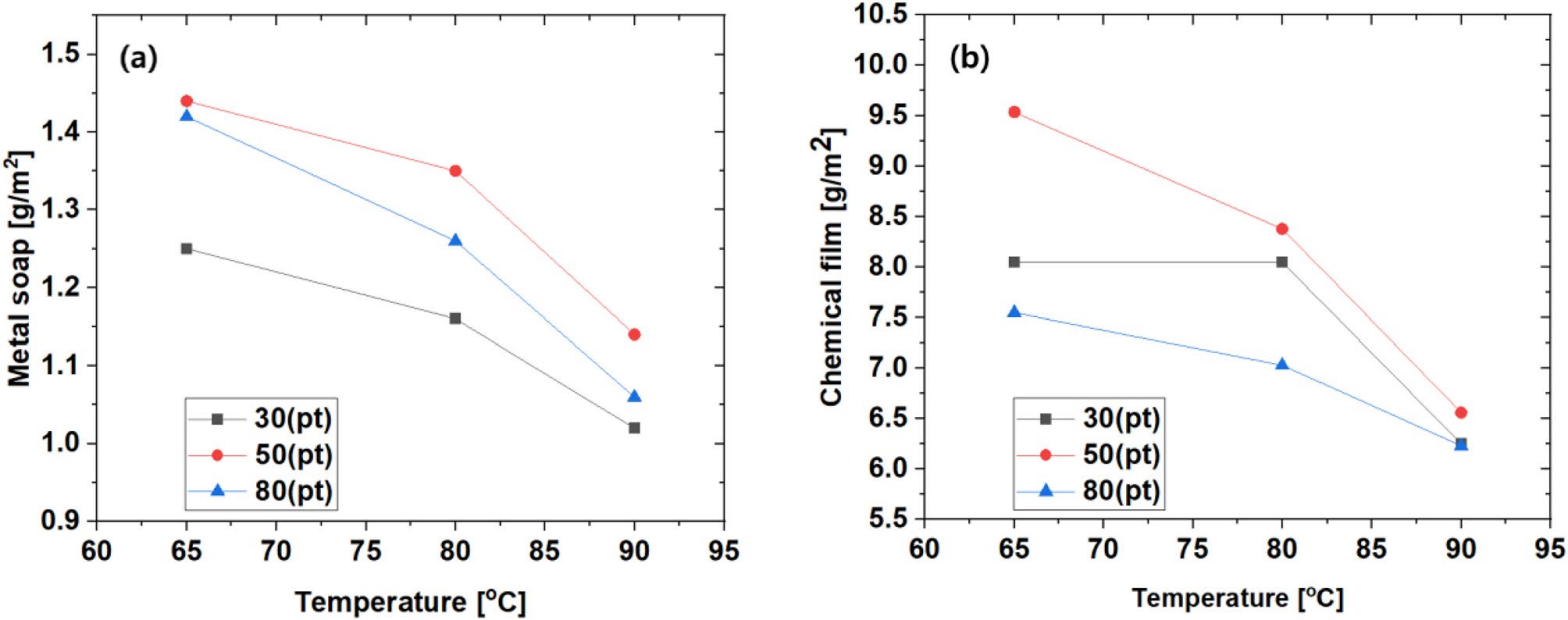
Relationship between (**a**) temperature and metal soap layer weight, (**b**) temperature and chemical film layer weight.

As described above, the phosphate coating layer is divided into a chemical conversion film layer, a metal soap layer, and an unreacted soap layer. To measure the weight of the unreacted soap layer, after measuring the weight (A) of the raw material, heat it in hot water for 30 min and measure the weight (B) again. The weight difference between A and B becomes the weight of the unreacted soap layer, which is converted into a weight value per unit area. Similarly, the weight (C) is re-measured after bathing in the G64 solution for 30 min, and the weight difference (B–C) is the weight of the metal soap layer. Finally, after measuring the weight (D) after heating in 5% Cr03 solution at 80 °C for 15 min, the weight difference (C-D) is determined as the weight of the chemical conversion film layer. Table 2 shows the weight per unit area of the film layer under each condition. Since the weight of the unreacted soap layer in Table 2 is an uncontrollable factor, the tendency of the weight of the metal soap and the chemical conversion coating layer was examined. As shown in Fig. 4a, the weight of the metal soap layer was the lowest when the phosphate solution concentration was 30pt, and the highest in the case of 50pt, so there was no proportional relationship between weight and concentration. However, it can be seen that the treatment temperature and the weight of the metal soap layer are inversely proportional. The tendency of the chemical conversion film layer weight is shown in Fig. 4b. Similarly to the metal soap layer, it tends to be inversely proportional to the treatment temperature, and it is possible to secure the most weight under the phosphate solution concentration condition of 50pt. However, unlike the metal soap layer, the weight was the least measured under the condition of 80pt phosphate treatment solution concentration. As a result, it is possible to secure the most weight of the metal soap and chemical conversion film layer at a concentration of 50pt of the phosphate treatment solution, and it can be seen that the lower the treatment temperature, the more advantageous it is in terms of securing the weight.

**Table 2:** Weight of phosphate coating layer.

Condition			Unreacted soap [g/m^2^]	Metal soap [g/m^2^]	Chemical film [g/m^2^]
Label	Concentration [pt]	Temp [°C]			
1C1T	30	65	8.81	1.25	8.05
1C2T	30	80	7.32	1.16	8.05
1C3T	30	90	6.91	1.02	6.25
2C1T	50	65	6.81	1.44	9.54
2C2T	50	80	9.32	1.35	8.38
2C3T	50	90	6.27	1.14	6.56
3C1T	80	65	11.36	1.42	7.55
3C2T	80	80	7.32	1.26	7.03
3C3T	80	90	5.89	1.06	6.23

## Repeated frictional test descriptions and result

**Figure 5:**
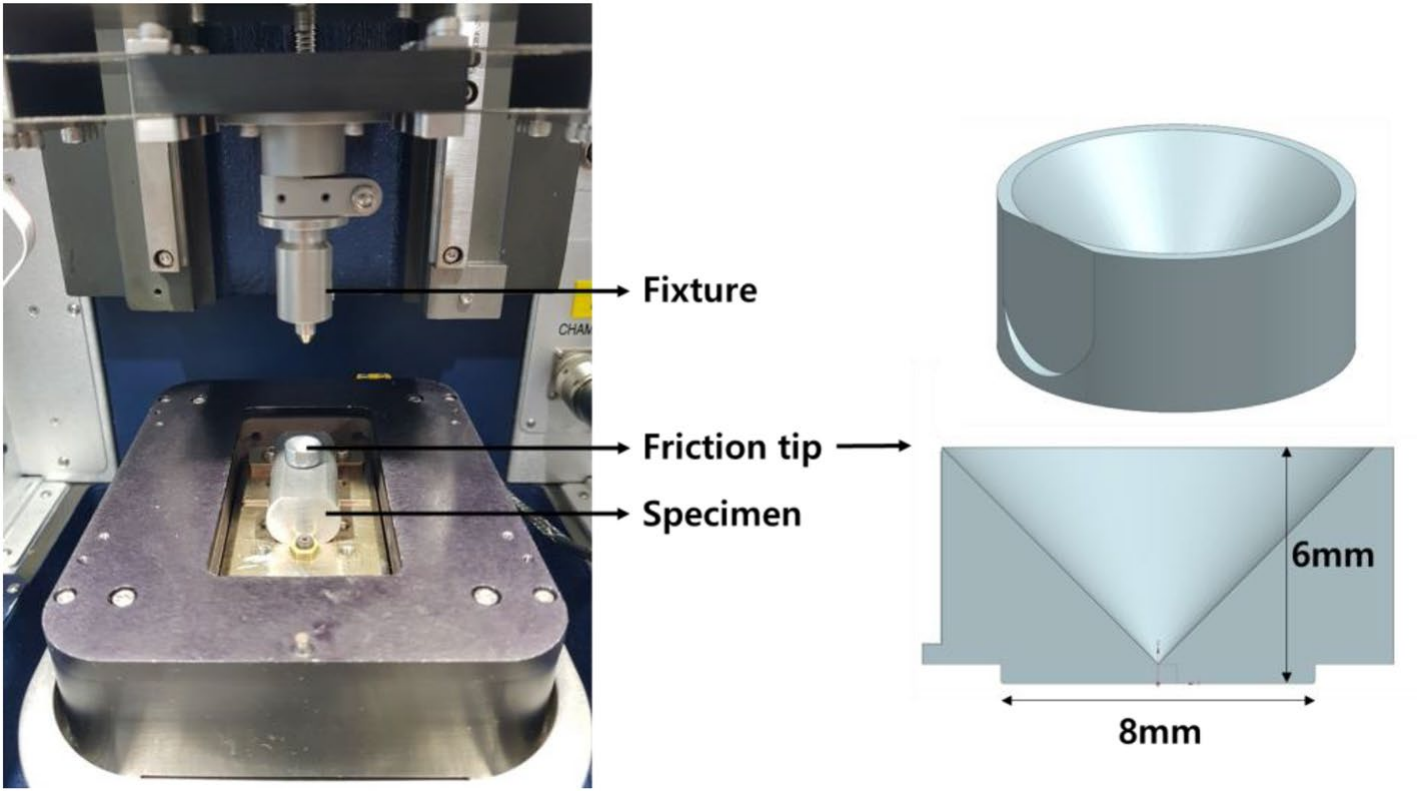
Composition of repeated friction test device.

**Figure 6:**
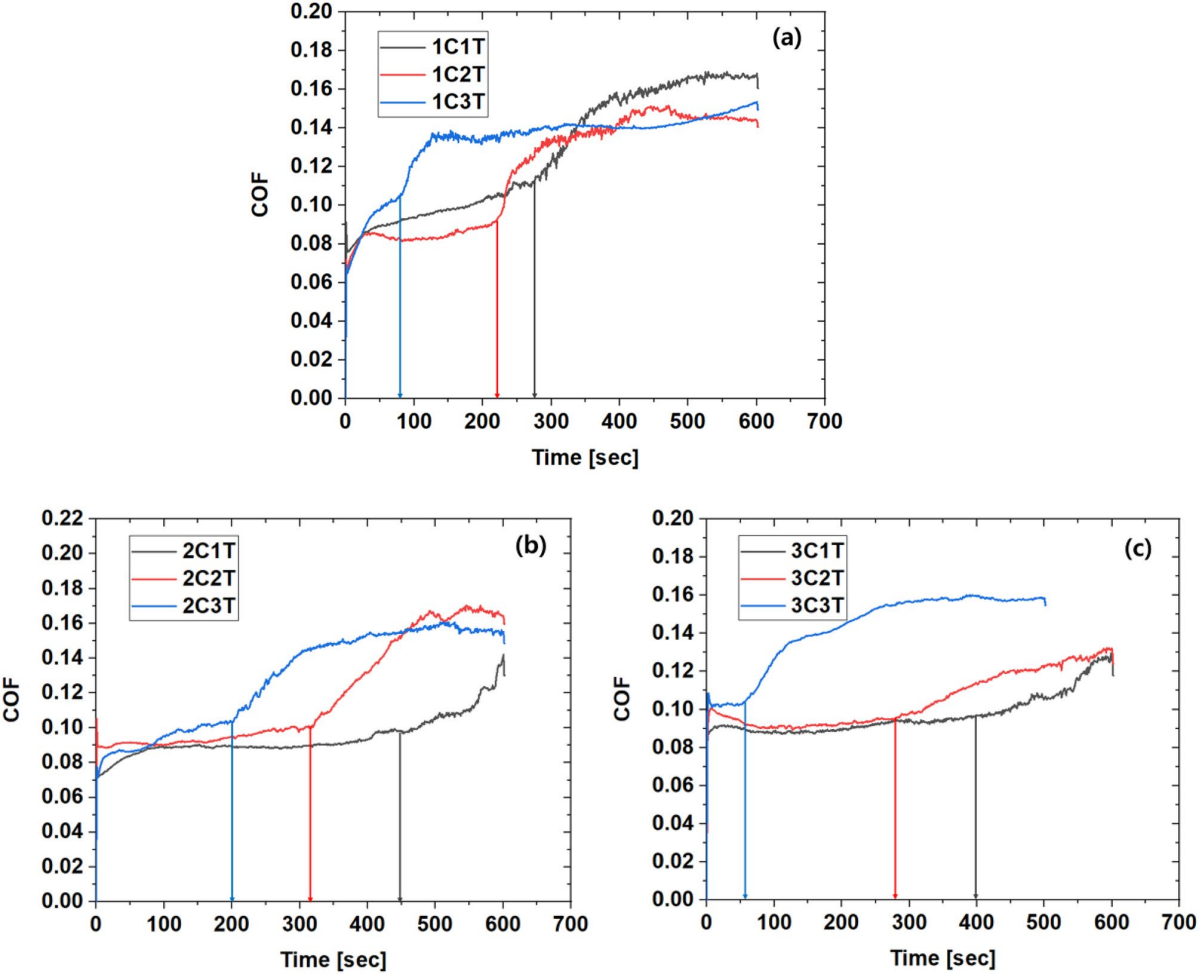
Friction coefficient behavior at (**a**) 30pt, (**b**) 50pt, (**c**) 80pt phosphating solution concentration.

As shown in Table 2, the phosphate coating treatment conditions were divided into a total of 9 cases, and a total of 4 repeated friction test specimens were prepared for each condition. As shown in Fig. 5, after fixing the test specimen to the bottom of the repeated friction test equipment, a friction tip with a diameter of 8 mm is placed in a flat section with a width of 10 mm. After the tip fixture at the top is accurately seated on the friction tip, a vertical load is applied to conduct the friction test. The material of the friction tip is SKD61, which is universally used for cold forging dies. The repeated friction test was carried out with a rate of 5 Hz means 3000 repetitions in 600 s on a round trip basis. Since the 5 mm stroke is on a one-way basis, the total stroke of the friction test is 30,000 mm. A vertical load of 300 N was applied, which corresponds to a pressure of about 5.97 MPa. Figure 6a shows the behavior of the friction coefficient according to the treatment temperature when the concentration of the phosphate treatment solution is 30pt. Initially, the peak between the two surfaces is offset, resulting in a temporarily high coefficient of friction^19^.

**Figure 7:**
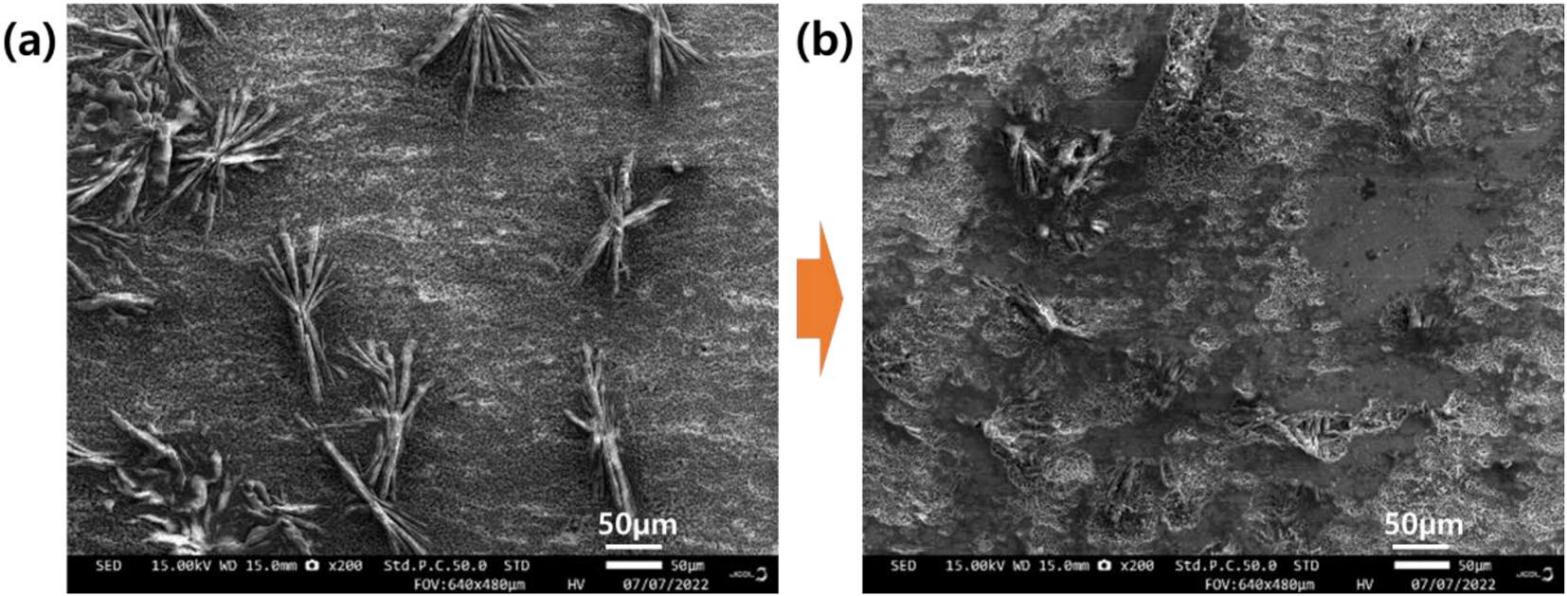
SEM measurement results (**a**) before and (**b**) after repeated friction test (Condition: 2C3T).

**Figure 8:**
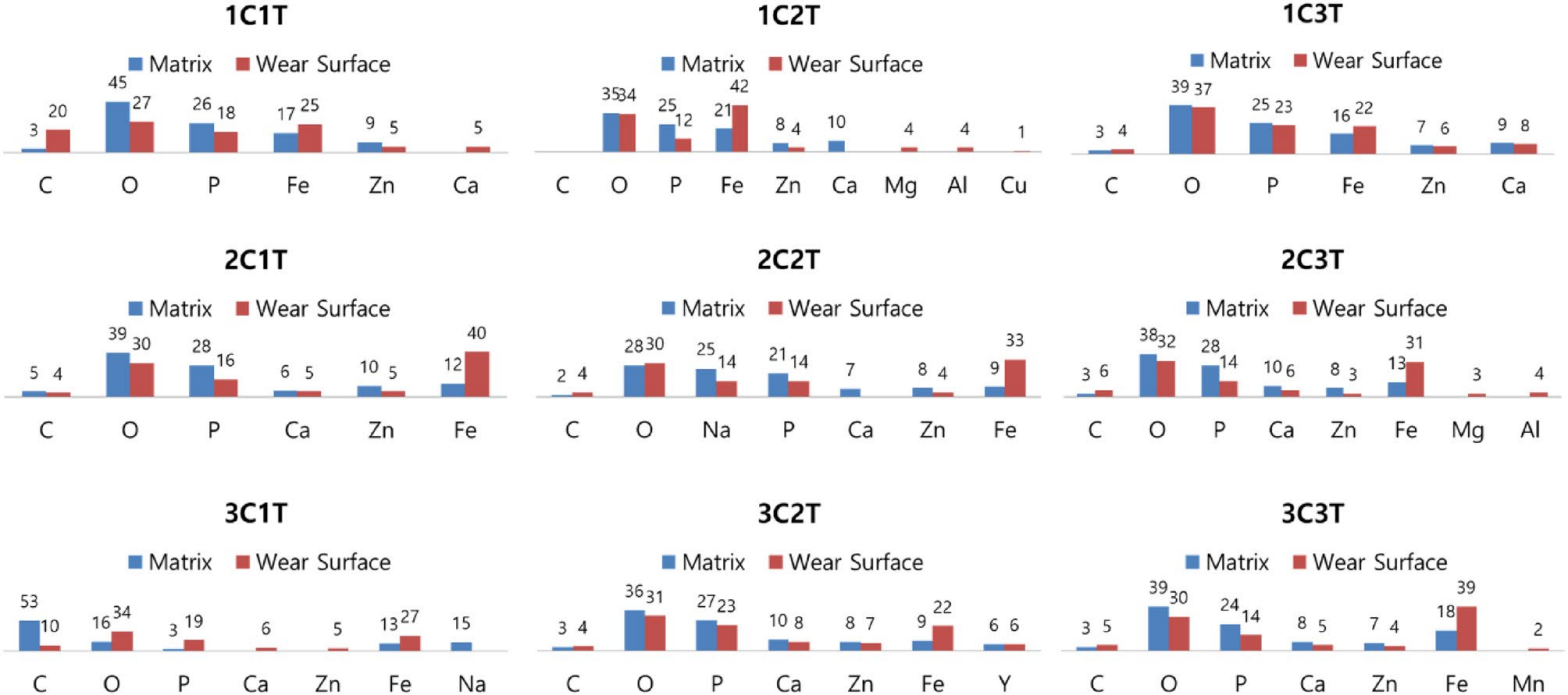
EDS measurement results before and after repeated friction test.

In all cases, the coefficient of friction is maintained at a certain level and then gradually increases. However, as the treatment temperature decreases– that is, as the weight of the phosphate film increases—the point at which the coefficient of friction increases tends to be delayed. Figures 6b and c show the friction coefficient behavior when the concentration of the phosphate treatment solution is 50 and 80pt. It shows a similar trend to the case of 30pt. After the friction coefficient was maintained up to a certain section, it tended to increase, and then entered the flat section again. As the repeated friction test begins, wear of the metal soap layer begins, and the coefficient of friction is maintained at a certain level. Afterwards, the transition period of the friction coefficient appears as the metal soap and the chemical conversion film are mixed. It is considered that the friction coefficient remains constant again after the metal soap layer is completely removed. The microstructure and components of the specimen before and after the friction test were analyzed using FE-SEM equipment (JSM-F100). Figure 7 shows the SEM measurement results of the non-friction surface and the friction surface of the repeated friction test specimen under the 2C3T condition, and Fig. 8 shows the EDS measurement results on the friction surface. Considering that P, Zn, and Ca were detected in all specimens, it is estimated that the chemical conversion film is not completely worn. Therefore, it is predicted that the friction coefficient will rise again as the die surface and the base material surface come into direct contact after the point at which the second flat section ends– that is, the point at which the chemical conversion film completely disappears.

The friction coefficient of the phosphate film layer for application to the FE simulation of the cold forging process was calculated. This can be done by dividing the metal soap layer and the chemical conversion film layer disappearance section, but since the chemical conversion film layer did not completely disappear in this experiment, only the friction coefficient of the metal soap layer and subsequent sections will be discussed. As shown in Figs. 6, the point at which the metal soap layer is eliminated in earnest is marked. In the 2C1T condition, where the weight of the phosphate film layer is the highest, the peeling of the metal soap layer starts the latest. Assuming that the material surface stabilized time was 20 s, the friction coefficient of the metal soap layer was defined as the average value from 20 s to the drop-off start time, and was calculated as shown in Table 3. It can be seen that the friction coefficient value for each condition is slightly different, but does not change significantly depending on the phosphate treatment condition. Next, the average value of the friction coefficient in the section after the metal soap layer started to drop off is also shown in Table 3. The friction coefficient continuously increases in the mixed section of the metal soap layer and the chemical conversion film layer, and the friction coefficient in the chemical conversion film layer is relatively larger than that of the metal soap layer. The reason why the friction coefficient increase rate is rather low in the 2C1T, 3C1T, and 3C2T conditions is that the peeling resistance of the metal soap layer is relatively high, and the peeling off section of the chemical conversion film layer is small. If the repeated friction test is conducted for more than 600 s, it is judged that the tendency will be similar to other conditions.

**Table 3:** Average coefficient of friction of metal soap layer.

Condition			Metal soap layer detaching time [sec]	COF_avg	
Label	Con. [pt]	Temp. [°C]		Before	After
1C1T	30	65	280	0.0984	0.1482
1C2T	30	80	210	0.0846	0.1341
1C3T	30	90	70	0.0953	0.1361
2C1T	50	65	450	0.0896	0.1103
2C2T	50	80	320	0.0942	0.1427
2C3T	50	90	200	0.0949	0.1424
3C1T	80	65	400	0.0913	0.1087
3C2T	80	80	280	0.0927	0.1108
3C3T	80	90	70	0.1039	0.1477

## Discussion on the limit life of automotive engine bolt die

**Figure 9:**
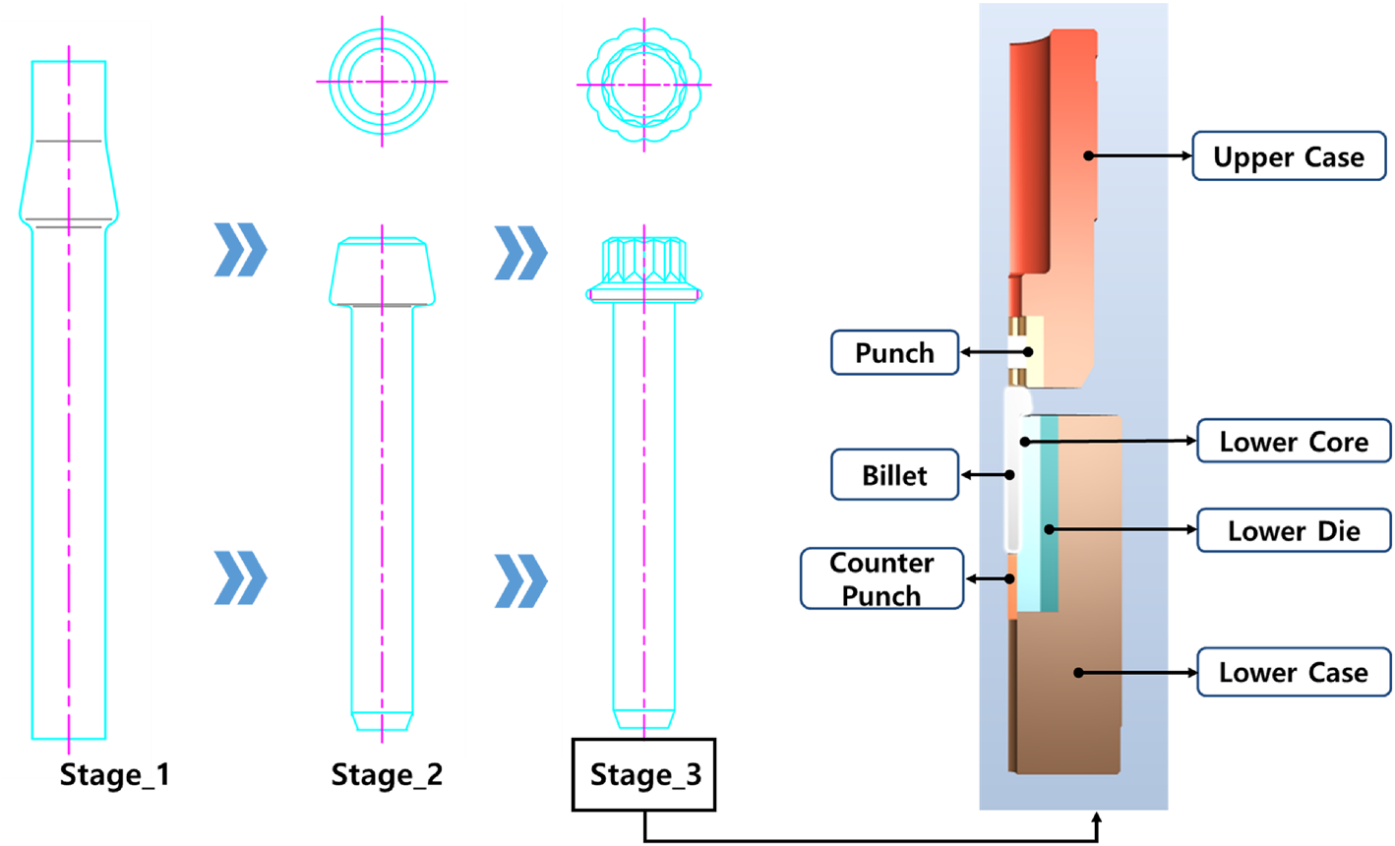
3D modeling for 3rd process of Connection Rod Bolt.

The strength of the connection rod bolts for automobile engines in Fig. 9 is about 1300 MPa, and high-strength 34CrMo4 material is applied. Due to its high strength, the problem of reducing die life cycle continues to occur due to friction between the material and the die in the multi-stage cold forming process. To evaluate the die life cycle according to the state of the phosphate film, FE modeling was performed according to the forging process. Steps 1 and 2 are 2D axisymmetric conditions, and step 3 is a 3D 1/4 model. Large plastic deformation occurs mainly in the head part, and it is predicted that the damage will occur primarily to the phosphate film. In particular, as the prismatic head part is formed in step 3, the stress acting on the upper die is greatly affected, which is highly likely to lead to premature breakage.

The limit life cycle of the upper punch in the 3rd step was predicted according to whether or not the phosphate coating was damaged. The procedure for predicting the quantitative life of a forging die is as follows^17^.

1. Prediction of the maximum principal stress acting on the forging die based on the forming simulation
2. Calculation of stress concentration factor (k_t_) and fatigue stress concentration factor (k_f_)
3. Conversion of maximum principal stress value (σ_analysis_) into fatigue stress (σ_fatigue_)
4. Calculation of die limit life cycle through substituting S–N diagram of fatigue stress

**Figure 10:**
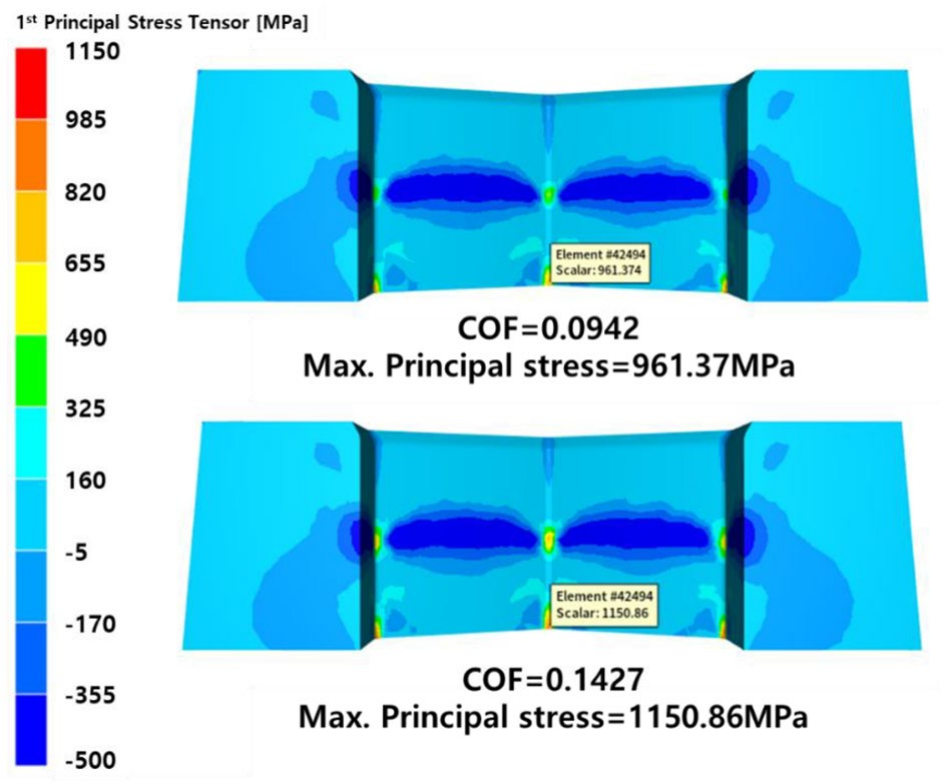
Comparison of maximum principal stress.

As such, the quantitative prediction result of the die life cycle depends on the accuracy of the prediction of maximum principal stress. COLDFORM NxT 2.2, a commercial analysis program, recommends a friction coefficient of 0.035 to 0.07 for phosphate coated materials. In general, phosphate film damage is not considered and a fixed friction coefficient is applied to the multi-stage cold forging process simulation. As shown in Fig. 6, the friction coefficient of the phosphate film in the initial state is 0.06 to 1.00, but since the friction coefficient gradually increases thereafter, it should be regarded as a variable. Figure 10 shows the results of predicting the maximum principal stress acting on the upper punch in the 3^rd^ step by applying the friction coefficient before and after the metal soap layer fell off under the 2C2T phosphate treatment condition, respectively. As a result of applying the friction coefficient before the removal of the metal soap layer, the maximum principal stress is 961 MPa, and when the friction coefficient after the removal is applied, the maximum principal stress rises to 1150 MPa. As a result of performing the above quantitative die life cycle prediction procedure, the die life cycle is 91,560 cycles / 23,212 cycles, respectively, and the die life cycle is reduced by 25.4% due to the metal soap layer falling off. Table 4 shows the die life cycle prediction results under all treatment conditions of different phosphate coatings.

**Table 4:** Die life cycle prediction results.

Condition			COF_avg / Die life cycle			
Label	Con. [pt]	Temp. [°C]	Before removal		After removal	
1C1T	30	65	0.0984	79,873	0.1482	20,181
1C2T	30	80	0.0846	109,607	0.1341	31,116
1C3T	30	90	0.0953	86,449	0.1361	28,950
2C1T	50	65	0.0896	104,526	0.1103	58,203
2C2T	50	80	0.0942	91,560	0.1427	23,212
2C3T	50	90	0.0949	89,229	0.1424	26,935
3C1T	80	65	0.0913	101,270	0.1087	62,995
3C2T	80	80	0.0927	98,115	0.1108	53,774
3C3T	80	90	0.1039	68,183	0.1477	17,467

**Figure 11:**
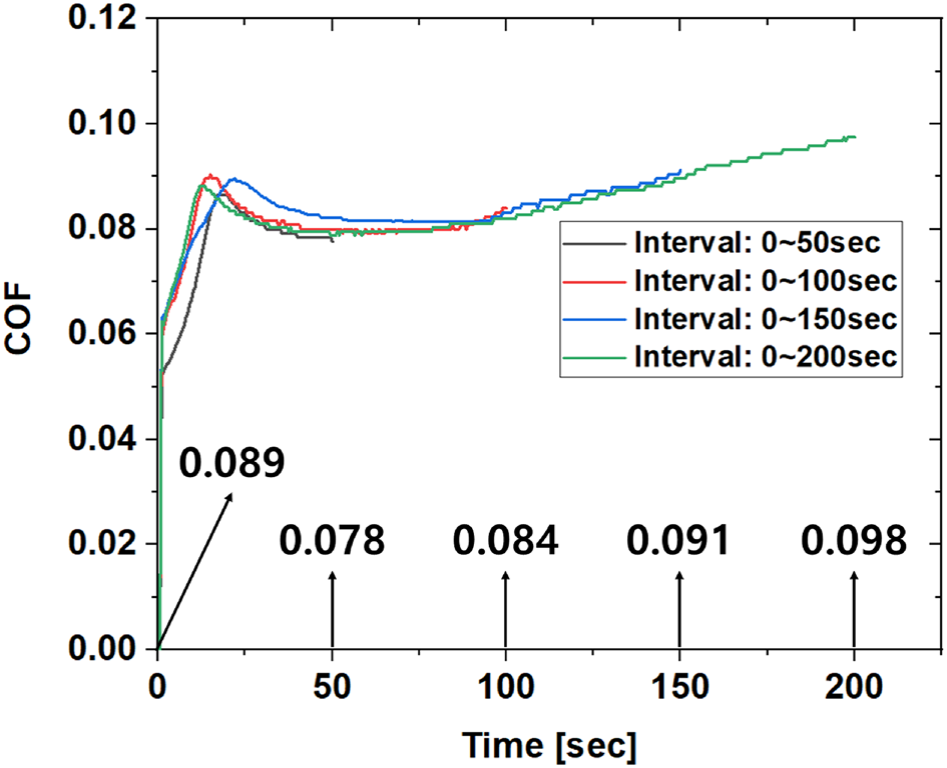
Friction test result according to interval subdivision.

The coefficient of friction continues to change slightly even in the section before the delamination of the metal soap layer. Therefore, by reflecting the change in the friction coefficient at each step in the multi-stage forging process, more accurate simulation and die life cycle prediction results can be obtained. Among the phosphate film treatment conditions in Table 4, the period before the drop-off of the metal soap layer that occurred under the 1C2T condition with the lowest friction coefficient was subdivided into 4 areas. Since the desorption point of the metal soap layer is about 210 s, additional experiments were conducted by dividing the section at 50 s intervals. As shown in Fig. 11, the behavior data of the friction coefficient was obtained. The friction coefficient in each section was defined as the friction coefficient value at the end of the repetition, and the friction coefficient of the undamaged material surface was defined as the average value of the friction coefficient corresponding to the first peak. The results are summarized in Fig. 11.

**Figure 12:**
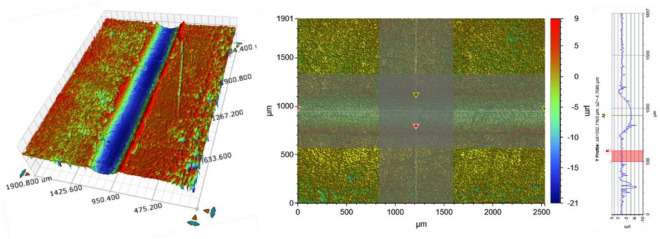
Example of 3D surface roughness measurement.

**Figure 13:**
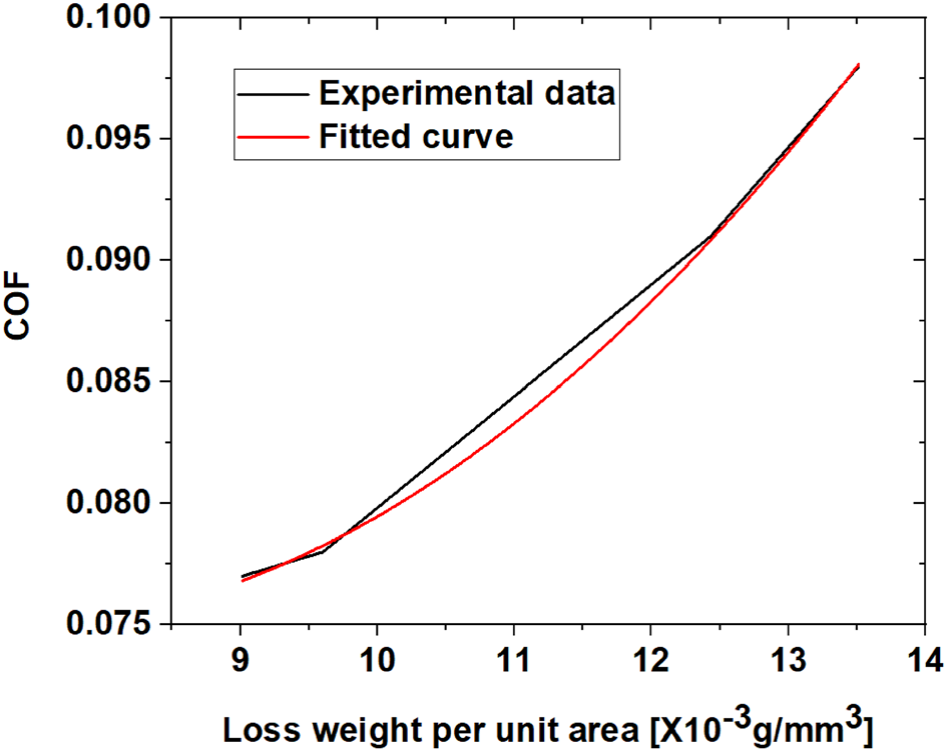
Relationship between phosphate film damage and coefficient of friction.

To quantitatively analyze the gradual damage of the phosphate film, the three-dimensional surface roughness of the friction surface of each specimen was measured. Bruker's Contour Gt-k equipment was used for 3-dimensional roughness measurement. The degree of damage was estimated according to the volume of the phosphate film lost by the repeated friction test. As shown in Fig. 12, the friction surface depth (D) and width (W) of all friction test specimens were measured. The cross-sectional profile scan was measured three times and the average value was used. Loss volume and loss weight were calculated based on the cross-sectional profile, and the results are summarized in Table 5. The density of the phosphate coating used for weight loss calculation is 2.769 g/mm^3^^20^. To calculate the friction coefficient in the multi-stage cold forging process of actual parts, it was converted into weight loss per unit area. In this process, the friction area was calculated using the damage width (W) and the total stroke. Figure 13 shows the relationship between the friction coefficient in Table 4 and the weight loss per unit area in Table 5.

**Table 5:** Friction coefficient according to number of repetitions.

Interval	Measurement [µm]		Loss volume [mm^3^]	Loss weight [g]	Weight per unit area [g/mm^3^]
	Depth	Width			
100	4.90	319.9	5225 × 10^–6^	14,421 × 10^–6^	9.016 × 10^–3^
200	5.05	330.4	5565 × 10^–6^	15,359 × 10^–6^	9.603 × 10^–3^
300	5.38	401.7	7205 × 10^–6^	19,885 × 10^–6^	12.432 × 10^–3^
400	5.61	148.6	7830 × 10^–6^	21,610 × 10^–6^	13.511 × 10^–3^

**Figure 14:**
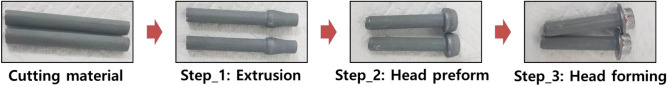
Con. Rod bolt sample according to forging process.

To apply the results of Fig. 13 to the connection rod bolt forming process, samples from each process were analyzed. In each step, the phosphate film is damaged, and as a result, the friction coefficient in the subsequent step changes. The total weight of the phosphate film of each sample in Fig. 14 was measured. The same process as the film weight measurement method of the frictional test specimen was performed, and the cumulative loss of the phosphate film per unit area was calculated as shown in Table 6. Based on the process design of the connection rod bolt, the total surface area after each process was completed was derived, and the cumulative loss per unit area was calculated by substituting this into the cumulative loss of the film. The total surface area of each process sample was derived based on the process design of the connection rod bolt, and the cumulative loss per unit area was calculated by substituting it into the cumulative loss of the film.

**Table 6:** Derivation of friction coefficient in Con. Rod bolt forming process.

Stage	Total film weight [g]	Loss film weight [g]	Weight per unit area [g/mm^3^]	COF
Initial	10.9	–	–	–
1st	9.1	1.8	6.398 × 10^–3^	0.089
2nd	8.2	2.7	9.597 × 10^–3^	0.078
3rd	7.3	3.6	12.796 × 10^–3^	0.094

The friction coefficient in each process was calculated as shown in Table 6 by substituting the cumulative loss per unit area of the phosphate film into the fitted polynomial equation. Since extrusion proceeds in the initial cutting state, 0.089, which corresponds to the number of repetitions of 0, is applied to the friction coefficient of step 1. At the time when the forging process in steps 1 and 2 is completed, weight loss of the phosphate film occurs, and it is possible to calculate the friction coefficient in the post-process. The forging simulation of the connection bolt was performed considering the change in the friction coefficient, and the die life cycle was evaluated. The die limit life prediction results in Table 4 are simply the results of applying the friction coefficient values before and after the removal of the metal soap layer of the phosphate film. On the other hand, if the friction coefficient at each step is calculated and applied as shown in Table 6, a more accurate prediction of the limit life is possible.

## Conclusion

In this study, we quantitatively analyzed the phosphate film damage of materials applied to the multi-stage cold forging process, and presented a guide that can be applied to a quantitative evaluation of the die life cycle.

1. The tendency of the coating weight formed on the surface of 34CrMo4 material under various phosphate coating treatment conditions was analyzed. It can be seen that the phosphate treatment solution concentration of 50pt and the low temperature treatment are advantageous for securing the weight.
2. In the repeated friction test, the behavior of the friction coefficient is divided into the metal soap layer section (maintenance), the mixed section (rising), and the conversion coating layer section (maintenance). It can be seen that the time at which the metal soap layer is removed is delayed as the film’s weight increases. In addition, by reflecting the friction coefficient behavior to FE simulation, prediction accuracy of formability and die life cycle can be improved.
3. By reflecting the behavior of the friction coefficient, the die life cycle prediction of the 3rd process of automobile engine bolts was performed. It can be seen that the die life cycle depends on the phosphate treatment conditions, and on whether or not the metal soap layer is removed. In designing the multi-stage cold forging process, the phosphate coating treatment condition and degree of damage are important factors.
4. The friction coefficient in the 1st to 3rd processes of the automobile engine bolt was predicted and applied to the FE simulation. The degree of film damage of the actual engine bolt sample was deduced based on the 3D roughness data, and the friction coefficient for each process was predicted in connection with the degree of damage of the repeated friction test specimen. It is possible to predict the die life cycle by applying the friction coefficient for each process, and it is judged that this will show better prediction accuracy than before.
5. Future research should pursue the creation of a universal mathematical model for the behavior of the friction coefficient according to the damage of the phosphate film.

## Data Availability

All data generated or analysed during this study are included in this published article.
